# Exploring the Demographics and Clinical Characteristics Related to the Expression of Angiotensin-Converting Enzyme 2, a Receptor of SARS-CoV-2

**DOI:** 10.3389/fmed.2020.00530

**Published:** 2020-08-19

**Authors:** Shengjie Li, Jianping Han, Aiping Zhang, Yi Han, Miaomiao Chen, Zhenzhen Liu, Mingxi Shao, Wenjun Cao

**Affiliations:** Clinical Laboratory, Eye & ENT Hospital, Fudan University, Shanghai, China

**Keywords:** coronavirus disease 2019, angiotensin covering enzyme II, susceptibility, gender, age, smoking, cancer

## Abstract

**Objective:** Coronavirus disease 2019 (COVID-19) was first reported in Wuhan, China, and has rapidly spread throughout the world. It has been reported that angiotensin-converting enzyme 2 (ACE2) is one of the major cellular entry receptors of SARS-CoV-2; thus, high ACE2 expression may increase susceptibility to infection. Therefore, we analyzed the expression of ACE2 in the blood to identify the individuals who may be susceptible to infection.

**Methods:** In total, 229 subjects were enrolled in this study, and reverse transcription-quantitative polymerase chain reaction and ELISA assay was used to identify the level of ACE2 mRNA expression and ACE2 protein level in the blood. Demographic and clinical characteristics, including age, gender, weight, height, smoking habits, drinking habits, diabetes, and hypertension, were obtained using a face-to-face questionnaire. Independent Student's *t*-test, Pearson's linear correlation, logistic regression analysis, and multiple linear regression correlation were performed to assess the association between these factors and the expression of ACE2.

**Results:** Higher level of ACE2 was observed in females, older subjects, subjects with hypertension, subjects with a cardiocerebrovascular disease, male smokers, and subjects with cancer (*p* < 0.05) than in other subjects. Multiple linear regression analysis showed that there is a statistically significant correlation between being a female and ACE2 expression (β = 0.550, *p* < 0.001), between older age and ACE2 expression (β = 0.197, *p* = 0.003), between smoking and ACE2 expression (β = 0.163, *p* = 0.037), and between cancer and ACE2 expression (β = 0.265, *p* < 0.001). Logistic regression analysis revealed that female subjects (odds ratio [OR] = 2.255, 95% confidence interval [CI] = 1.770–2.872), subjects with hypertension (OR = 1.264, 95% CI = 1.075–1.486), subjects with a cardiocerebrovascular disease (OR = 1.271, 95% CI = 1.023–1.579), subjects with cancer (OR = 1.695, 95% CI = 1.253–2.293), and subjects above 60 years of age (OR = 3.097, 95% CI = 1.078–8.896) are at an increased risk of infection due to their high expression of ACE2.

**Conclusion:** The level of ACE2 is higher in females, older subjects, smokers, and subjects with cancer than in other subjects, indicating that some of which are at higher risk for the severe forms of COVID-19 when they are exposed to the SARS-Cov-2.

## Introduction

In December 2019, coronavirus disease 2019 (COVID-19) was detected in patients in Wuhan, Hubei Province, China (1). The virus started spreading rapidly throughout China and the world (2, 3). According to the World Health Organization, as of June 10, 2020, 7,145,539 laboratory-confirmed cases were detected, with a death toll of 408,025 patients (4). Given the rapid spreading of this outbreak, it is urgent to identify subjects who may be susceptible to infection and to further control the spread of the disease to those susceptible subjects.

It has been shown from severe acute respiratory syndrome coronavirus (SARS-Cov) and Middle East respiratory syndrome coronavirus (MERS-CoV) that humans exhibit disparities in susceptibility to these viruses (5–7). For example, Liu et al. (5) reported that older age (odds ratio [OR] = 8.546, 95% confidence interval [CI] = 1.628–44.864, *p* = 0.011) and smoking (OR = 14.285, 95% CI = 1.577–25.000, *p* = 0.018) are risk factors for the progression of COVID-19. Rao et al. (8) showed that over 25% of patients with COVID-19 have a history of hypertension (12.9%) and diabetes (5.4%). However, it is still unclear whether the above factors, or perhaps even other factors, are associated with susceptibility to COVID-19. It has been reported that angiotensin-converting enzyme 2 (ACE2) is one of the major cellular entry receptors of COVID-19 (9), indicating that a higher expression of ACE2 may lead to increased susceptibility to infection. Several studies have investigated the relationship between the expression level of ACE2 and the demographic or clinical characteristics of COVID-19. For example, Rao et al. performed a phenome-wide Mendelian randomization study and found that type II diabetes is causally linked to an increased expression of ACE2 (8). Chen et al. (10) showed that ACE2 is mainly expressed in the epithelial cells of the colon and that its expression is increased the most in patients with colorectal cancer followed by patients with adenoma, compared to healthy controls. Moreover, Cai (11) reported that ACE2 expression is significantly higher in the lungs of former smokers than in non-smokers. According to previous evidence, the expression level of ACE2 is associated with susceptibility to COVID-19 infection.

However, previous studies had several limitations. On the one hand, most of the samples were derived from different types of tissues, such as lung and colon tissues, which may not be fully reflective of the expression in the whole body. Another potential limitation was that the sample size was too small to draw conclusions. Therefore, in this study, we performed a cross-sectional study to explore the clinical/demographic characteristics that may lead to an increased expression of ACE2, which may in turn result in greater susceptibility to infection with COVID-19 when they are exposed to the SARS-Cov-2.

## Materials and Methods

### Subjects

This study was conducted at the Department of clinical laboratory, Eye, Ear, Nose and Throat (Eye and ENT) Hospital of Fudan University, Shanghai, China, and was approved by the Ethics Committee of the same hospital. This study adhered to the principles of the Declaration of Helsinki. Informed consent was obtained from all subjects. All subjects were recruited from the Eye and ENT Hospital of Fudan University.

### Examination

Medical examinations, including the assessment of electrocardiograms, X-rays, liver function, blood glucose, infectious diseases, renal function, blood pressure, heart rate, body temperature, height, and weight, were performed for all subjects by the respective specialty physicians at the Eye and ENT Hospital of Fudan University. Demographic and clinical characteristics, including age, gender, weight, height, smoking habits, drinking habits, diabetes, and hypertension, were collected using a face-to-face questionnaire. Body mass index (BMI) was calculated as the weight in kilograms divided by the height in meters squared. Drinking was defined as more than three drinks per week for more than 6 months (current or former), and smoking was defined as more than one cigarette per day for more than 6 months (current or former) (12).

The exclusion criteria were as follows: patients with an autoimmune disease, patients with an acute infectious disease, patients with a metabolic syndrome, patients who have undergone surgery within the previous 2 months, patients with abnormal hepatic or renal function, patients with a hereditary disease, and patients whose body temperature was above 37.5°C. Hence, a total of 20 subjects (surgery = 5, metabolic syndrome = 5, autoimmune disease = 4, acute infectious disease = 4, and hereditary disease = 2) were excluded.

### RNA Isolation and Detection

Blood samples were obtained in the morning after subjects had fasted for 8 h via standard venipuncture in the antecubital fossae (anterior elbow veins). First, blood samples (2 mL) were collected in ethylenediaminetetraacetic acid (EDTA) tubes. Total RNA was extracted using a TRIzol reagent (Sigma-Aldrich, Merck KGaA, Darmstadt, Germany) as per the manufacturer's instructions. The quality and integrity of the acquired total RNA were evaluated using a NanoDrop™ 2000c (Thermo Fisher Scientific, Inc., Wilmington, DE, USA). For reverse transcription-quantitative polymerase chain reaction (RT-qPCR), 1,000 ng of total RNA was reverse-transcribed with 2 μL of 5X OneStep RT Mix. The RT-qPCR reaction was performed using 1 μL of RT products, 0.2 μL of 10 μM forward primer, 0.2 μL of 10 μM pmol reverse primer, and 5 μL of 2X SYBR Green I qPCR mix and completed to 10 μL with nuclease-free water. The primers used were as follows: ACE2-forward, 5′-AAAGGAACAGTCCACACTTGCCC-3′, and ACE2-reverse, 5′-TGAAGACCCATTTTGCTGAAGAGCC-3′.

### ELISA Assay

The protein level of ACE2 was further measured by ELISA kit. The serum samples were subjected to ACE2 assay as described in the ACE2 assay kit (ab235649, Abcam, USA). The ACE2 concentration of each sample was detected by multimode microplate readers (Biotek SynergyH1, USA) at 450 nm.

### Statistical Analysis

All analyses were performed using the Statistical Package for the Social Sciences software, version 13.0 (SPSS Inc., Chicago, IL, USA). Figures were created using GraphPad Prism 6 (GraphPad Software, La Jolla, CA, USA). Results are presented as mean ± standard deviation (SD). Normality was assessed using the Kolmogorov–Smirnoff test. An independent Student's *t*-test, Mann-Whitney *U*-test, Pearson's analysis, and one-way analysis of variance (ANOVA) were used. Multivariate linear regression analysis was performed to evaluate the association between ACE2 levels and factors. Logistic regression analysis was performed to estimate the ORs with 95% CIs. A *p* < 0.05 was considered statistically significant.

## Results

### Characteristics of the Study Patients

A total of 229 subjects (125 males, 104 females) were enrolled, which was conducted at the Eye and ENT Hospital of Fudan University. The mean age of the males and females was 51.94 ± 17.59 years and 51.22 ± 16.96 years, respectively. Among all subjects, the proportion of smoking history (37.6 vs. 4.81%) and drinking history (20.8 vs. 2.88%) was significantly higher in male subjects than in female subjects. Moreover, the prevalence of diabetes, hypertension, cardiocerebrovascular diseases, and cancer was similar between male and female subjects. The demographic and clinical characteristics of the subjects are shown in Table 1.

**Table 1:** Demographics and clinical characteristics.

	**Number of subjects/Mean value**
Gender, male/female	125/104
**Age, years**	
Mean ± SD, male/female	51.94 ± 17.59/51.22 ± 16.96
0–20, male/female	9, 5/4
20–40, male/female	54, 29/25
40–60, male/female	76, 44/32
>60, male/female	90, 47/43
**BMI, mean, Kg/m**^**2**^	
Mean ± SD, male	24.55 ± 3.77
Mean ± SD, female	22.61 ± 3.67
**Smoking habit, proportion% (yes)**	
Male	37.60 (47)
Female	4.81 (5)
**Drinking habit, proportion% (yes)**	
Male	20.80 (26)
Female	2.88 (3)
**Diabetes mellitus, proportion% (yes)**	
Male	15.20 (19)
Female	11.54 (12)
**Hypertension, proportion% (yes)**	
Male	26.40 (33)
Female	36.54 (38)
**Cardio-cerebrovascular disease, proportion% (yes)**	
Male	9.60 (12)
Female	11.54 (12)
**Cancer, proportion% (yes)**	
Male	4.00 (5)
Female	5.77 (6)

### Comparison of ACE2 mRNA and Protein Levels in Subjects With Different Demographic and Clinical Characteristics

**Figure 1:**
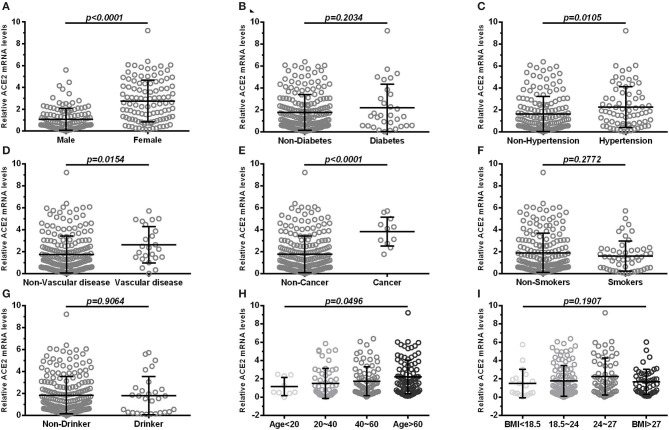
Comparison of ACE2 mRNA levels in subjects with different demographic and clinical characteristics **(A)** shows expression in blood of male and female; **(B)** shows expression in blood of diabetes and non-diabetes; **(C)** shows expression in blood of hypertension and non-hypertension; **(D)** shows expression in blood of vascular disease and non-vascular disease; **(E)** shows expression in blood of cancer and non-cancer; **(F)** shows expression in blood of smokers and non-smokers; **(G)** shows expression in blood of drinker and non-drinker; **(H,I)** shows groups in age (<20, 20–40, 40–60, >60) and BMI (<18.5, 18.5–24, 24–27, >27). Vascular, cardiocerebrovascular disease; BMI, body mass index. Each data point represents one subject. Data are expressed as mean ± SD. Independent Student's *t*-test and Mann–Whitney *U*-test were used.

ACE2 expression was higher in female subjects than in male subjects (*p* < 0.0001; Figure 1A). There was no significant differences (*p* > 0.05) in the expression of ACE2 between subjects with diabetes and subjects without (Figure 1B). Furthermore, subjects with hypertension (Figure 1C), cardiocerebrovascular diseases (Figure 1D), and cancer (Figure 1E) exhibited a higher ACE2 expression than that of those not suffering from these diseases (both *p* < 0.05). Moreover, we observed no significant differences (*p* > 0.05) in the expression of ACE2 between smokers and non-smokers (Figure 1F), and between drinkers and non-drinkers (Figure 1G). The expression of ACE2 was lowest in the >60 age group followed by the 40–60, 20–40, and <20 age groups, and the differences were statistically significant (*p* = 0.0496; Figure 1H). There was no significant differences (*p* > 0.05) in the expression of ACE2 among BMI subgroup (Figure 1I). A similar result was observed when ACE2 protein levels were compared in subjects with different demographic and clinical characteristics (Table 2).

**Table 2:** Comparison of ACE2 protein levels in subjects with different demographic and clinical characteristics.

	**ACE2**	***t*-value**	***P*-value**
**Gender**			
Male	16.28 ± 5.87		
Female	2,009 ± 5.63	4.983	<0.001
**Diabetes**			
Yes	19.63 ± 6.15		
No	17.75 ± 6.02	1.609	0.109
**Hypertension**			
Yes	20.24 ± 5.54		
No	17.01 ± 6.02	3.956	<0.001
**Vascular disease**			
Yes	21.97 ± 5.58		
No	17.53 ± 5.94	3.477	0.001
**Cancer**			
Yes	27.52 ± 2.44		
No	17.52 ± 5.78	11.977	<0.001
**Smoking**			
Yes	19.65 ± 5.77		
No	17.52 ± 6.07	2.318	0.023
**Drinking**			
Yes	18.81 ± 7.85		
No	17.88 ± 5.76	0.612	0.545
**Age, years**			
<20	15.67 ± 6.31		
20–40	15.94 ± 7.09		
40–60	18.51 ± 5.58		
>60	19.05 ± 5.44	3.733	0.012
**BMI, Kg/M**^**2**^			
<18.5	16.69 ± 5.88		
18.5–24	17.98 ± 6.14		
24–27	18.34 ± 6.16		
>27	18.08 ± 6.07	0.323	0.809

### Comparison of ACE2 mRNA and Protein Levels in Subjects With Different Demographic and Clinical Characteristics, Stratified According to Sex

**Figure 2:**
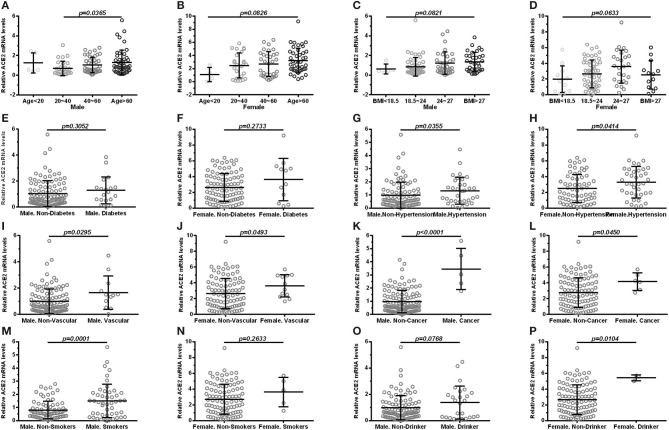
Comparison of ACE2 mRNA levels in subjects with different demographic and clinical characteristics, stratified according to sex. (male: **A**, **C**, **E**, **G**, **I**, **K**, **M**, **O**; female: **B**, **D**, **F**, **H**, **J**, **L**, **N**, **P**). **(A,B)** shows groups in age (<20, 20–40, 40–60, >60); **(C,D)** shows groups in BMI (<18.5, 18.5–24, 24–27, >27); **(E,F)** shows expression in diabetes and non-diabetes; **(G,H)** shows expression in hypertension and non-hypertension; **(I,J)** shows expression in vascular disease and non-vascular disease; **(K,L)** shows expression in cancer and non-cancer; **(M,N)** shows expression in smokers and non-smokers; **(O,P)** shows expression in drinker and non-drinker. Vascular, cardiocerebrovascular disease; BMI, body mass index. Each data point represents one subject. Data are expressed as mean ± SD. One-way ANOVA and Mann–Whitney *U*-test were used.

According to sex, all subjects were divided into male and female subgroups. The expression of ACE2 was found to be lowest in the >60 age group followed by the 40–60, 20–40, and <20 age groups in both males (Figure 2A) and females (Figure 2B). Moreover, we observed no significant differences (*p* > 0.05) in the expression of ACE2 among BMI subgroup (Figures 2C,D), and between subjects with diabetes and subjects without (Figures 2E,F) in the male and female subgroup. In both male and female subgroups, a higher ACE2 expression was observed in subjects with hypertension (Figures 2G,H), cardiocerebrovascular diseases (Figures 2I,J), and cancer (Figures 2K,L), with *p* < 0.05. Male smokers exhibited a significantly higher ACE2 expression (*p* = 0.0001; Figure 2M) compared to male non-smokers, but not in females (*p* = 0.2633; Figure 2N). Interestingly, female drinkers exhibited a higher ACE2 expression compared to non-drinkers (*p* = 0.0104; Figure 2P), but not in males (Figure 2O). A similar result was observed when ACE2 protein levels were compared in subjects with different demographic and clinical characteristics, stratified according to sex (Table 3).

**Table 3:** Comparison of ACE2 protein levels in subjects with different demographic and clinical characteristics, stratified according to sex.

	**Male**	***P*-value**	**Female**	***P*-value**
**Diabetes**				
Yes	18.15 ± 5.12		21.95 ± 7.12	
No	15.94 ± 5.95	0.133	19.84 ± 5.40	0.224
**Hypertension**				
Yes	18.99 ± 5.27		21.59 ± 5.44	
No	15.31 ± 5.79	0.002	19.22 ± 5.57	0.035
**Vascular disease**				
Yes	21.21 ± 6.47		22.79 ± 4.50	
No	15.76 ± 5.58	0.002	19.75 ± 5.68	0.041
**Cancer**				
Yes	28.40 ± 2.67		26.78 ± 2.17	
No	15.78 ± 5.41	<0.001	19.73 ± 5.51	0.002
**Smoking**				
Yes	19.14 ± 5.72		24.43 ± 4.02	
No	14.56 ± 5.28	<0.001	19.91 ± 5.61	0.079
**Drinking**				
Yes	17.96 ± 7.77		26.18 ± 4.05	
No	15.84 ± 5.22	0.198	19.95 ± 5.57	0.109
**Age, years**				
<20	16.60 ± 4.87		14.49 ± 8.45	
20–40	14.14 ± 6.45		18.03 ± 7.35	
40–60	16.72 ± 5.61		20.32 ± 4.99	
>60	17.20 ± 5.67	0.030	21.73 ± 3.87	0.010
**BMI, Kg/M**^**2**^				
<18.5	13.17 ± 1.76		18.16 ± 6.05	
18.5–24	15.33 ± 6.02		20.71 ± 5.02	
24–27	16.55 ± 5.48		20.61 ± 6.12	
>27	17.87 ± 6.14	0.159	18.61 ± 6.46	0.370

**Figure 3:**
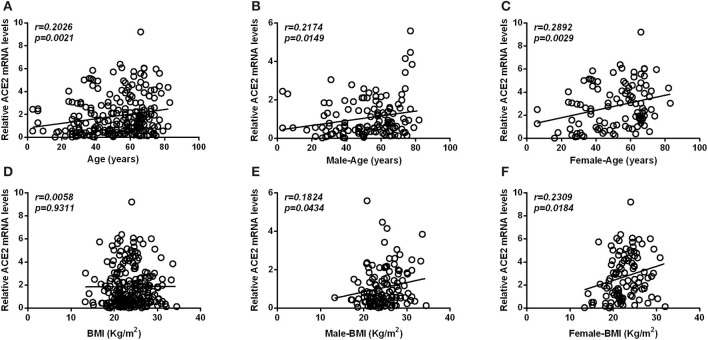
Scatterplot of patient individual measurements for age and BMI vs. ACE2 mRNA levels. **(A–C)** Shows the association between ACE2 mRNA levels and age, between ACE2 mRNA levels and age in male subjects, and between ACE2 mRNA levels and age in female subjects. **(D–F)** Shows the association between ACE2 mRNA levels and BMI, between ACE2 mRNA levels and BMI in male subjects and between ACE2 mRNA levels and BMI in female subjects. Each data point represents one patient. Pearson's analysis was used.

Pearson's analysis also showed that there was a significantly positive correlation between age and the expression level of ACE2 (Figure 3A), in both males (Figure 3B) and females (Figure 3C). Although we observed no significant association between BMI and the expression level of ACE2 in any of the subjects (Figure 3D), a significant correlation was also found in both male (Figure 3E) and female subgroup (Figure 3F). Furthermore, there was a significantly positive correlation between ACE2 mRNA levels and ACE2 protein levels (*r* = 0.677, *p* < 0.001).

### Multiple Linear Regression for the Association Between Age, Gender, Smoking Habits, and Cancer and ACE2 mRNA Levels

After adjusting for age, sex, BMI, hypertension, diabetes, drinking habits, smoking habits, cardiocerebrovascular diseases, and cancer, multiple linear regression analysis showed that there is a statistically significant correlation between being a female and ACE2 expression (β = 0.550, *p* < 0.001), between older age and ACE2 expression (β = 0.197, *p* = 0.003), between smoking and ACE2 expression (β = 0.163, *p* = 0.037), and between cancer and ACE2 expression (β = 0.265, *p* < 0.001). In the sex-stratified subgroup, adjusting for age, BMI, hypertension, diabetes, drinking habits, smoking habits, cardiocerebrovascular diseases, and cancer, similar results were also observed in the male and female subgroups. However, a relationship between smoking and ACE2 expression was just observed in the male subgroup, but not in the female subgroup (see Table 4 for details).

**Table 4:** Multiple linear correlation analysis to assess the relationship between age, gender, smoking, and cancer with ACE2 mRNA levels.

	**Multiple linear correlation**		**Multiple linear correlation-Male**		**Multiple linear correlation-Female**	
	**Beta**	***P* (95%CI)**	**Beta**	***P* (95%CI)**	**Beta**	***P* (95%CI)**
Female	0.550	<0.001 (1.473–2.310)	NA	NA	NA	NA
Older age	0.197	0.003 (0.007–0.032)	0.162	0.038 (0.001–0.018)	0.318	0.001 (0.015–0.057)
Smoking	0.163	0.037 (0.040–0.299)	0.184	0.043 (0.012–0.754)	NA	NA
Cancer	0.265	<0.001 (1.031–3.192)	0.477	<0.001 (1.650–3.203)	0.220	0.020 (0.283–3.296)

*Beta, standardized coefficients*.

### Logistic Regression Analysis to Identify the Risk Factors for the ACE2 mRNA Levels

After adjusting for age, sex, BMI, hypertension, diabetes, drinking habits, smoking habits, cardiocerebrovascular diseases, and cancer, logistic regression analysis revealed that females (OR = 2.255, 95% CI = 1.770–2.872), subjects with hypertension (OR = 1.264, 95% CI =1.075–1.486), subjects with cardiocerebrovascular diseases (OR = 1.271, 95% CI = 1.023–1.579), subjects with cancer (OR = 1.695, 95% CI = 1.253–2.293), and subjects above 60 years of age (OR = 3.097, 95% CI = 1.078–8.896) are at an increased risk for infection due to their high expression of ACE2. In both male and female subgroups, after adjusting for age, BMI, hypertension, diabetes, drinking habits, smoking habits, cardiocerebrovascular diseases, and cancer, similar results were also observed. Moreover, male smokers (OR = 1.710, 95% CI = 1.149–2.544) were found to be at an increased risk due to their high expression of ACE2, but not female subjects (see Table 5 for details).

**Table 5:** Logistic regression analyses to identify risk factors for the ACE2 mRNA levels.

	**Logistic regression**		**Logistic regression (Male)**		**Logistic regression (Female)**	
	**OR**	**P (95%CI)**	**OR**	**P (95%CI)**	**OR**	**P (95%CI)**
**Gender**						
Male	1		NA	NA	NA	NA
Female	2.255	<0.001 (1.770–2.872)	NA	NA	NA	NA
**Smoking**						
No	1		1		1	
Yes	0.888	0.235 (0.730–1.080)	1.710	0.008 (1.149–2.544)	0.889	0.244 (0.729–1.084)
**Drinking**						
No	1		1		1	
Yes	0.977	0.847 (0.775–1.233)	1.414	0.086 (0.953–2.100)	0.967	0.776 (0.765–1.221)
**Diabetes**						
No	1		1		1	
Yes	1.182	0.105 (0.966–1.446)	1.256	0.307 (0.811–1.947)	1.180	0.112 (0.962–1.448)
**Hypertension**						
No	1		1		1	
Yes	1.264	0.005 (1.075–1.486)	1.845	0.003 (1.226–2.776)	1.254	0.006 (1.066–1.475)
**Vascular**						
No	1		1		1	
Yes	1.271	0.031 (1.023–1.579)	1.946	0.006 (1.208–3.137)	1.276	0.031 (1.023–1.591)
**Cancer**						
No	1		1		1	
Yes	1.695	0.001 (1.253–2.293)	4.720	0.001 (1.900–11.633)	1.685	0.001 (1.245–2.281)
**Age**						
<20	1		1		1	
20–40	1.175	0.539 (0.702–1.967)	2.802	0.378 (0.284–27.623)	1.758	0.186 (0.762–4.060)
40–60	2.365	0.094 (0.863–6.482)	10.370	0.144 (0.450–238.776)	2.086	0.153 (0.761–5.716)
>60	3.097	0.036 (1.078–8.896)	11.571	0.107 (0.592–226.323)	4.027	0.049 (1.003–16.176)
**BMI**						
<18.5	1		1		1	
18.5–24	1.074	0.667 (0.776–1.487)	1.46	0.611 (0.355–6.433)	1.278	0.222 (0.862–1.895)
24–27	1.245	0.204 (0.888–1.746)	2.427	0.282 (0.483–12.203)	1.532	0.067 (0.970–2.419)
>27	1.056	0.794 (0.702–1.588)	3.440	0.151 (0.636–18.596)	1.222	0.411 (0.785–1.972)

*Vascular, cardiocerebrovascular disease; BMI, body mass index*.

## Discussion

As far as we know, previous studies failed to comprehensively study many different risk factors/diseases and evaluate whether these are risk factors for COVID-19. In this study, we investigated the disparities related to age, gender, BMI, smoking habits, drinking habits, diabetes, hypertension, cardiocerebrovascular diseases, and cancer in ACE2 gene expression, which in turn may influence susceptibility to infection with COVID-19.

From our analysis, the most credible finding was the link between gender, age, smoking habits (male subjects), and cancer and the expression of ACE2, which was supported by an independent Student's *t*-test, multivariate linear regression analysis, and logistic regression analysis. Other results are preliminary but are worthy of further studies. For example, hypertension and cardiocerebrovascular diseases were found to cause a higher expression of ACE2, and showed positive associations with the expression of ACE2.

Numerous studies showed that older age is associated with susceptibility to infection and the presence of a primary composite endpoint of COVID-19 infection (admission to an intensive care unit [ICU], the use of mechanical ventilation, or death) (3, 6, 13–15). For example, Zhang et al. (7) reported that 70% of the COVID-19 patients were age>50 years older. Chen et al. (13) performed a retrospective, single-center study in Shanghai, China, and reported that age (OR = 1.06) is independently associated with admission to the ICU. Wu et al. (14) reported that older age (hazard ratio [HR] = 3.26, 95% CI = 2.08–5.11; HR = 6.17, 95% CI = 3.26–11.67, respectively), is a risk factor associated with the development of COVID-19 and eventually death. We found that the expression of ACE2 was highest in the >60 age group followed by the 40–60, 20–40, and <20 age groups. Moreover, Pearson's analysis, multivariate linear regression analysis, and logistic regression analysis also revealed that there is a statistically significant positive correlation between age and the expression of ACE2. This may explain the reason why there is an association between being older and susceptibility to infection with COVID-19 and the presence of a primary composite endpoint.

In addition, we found that smokers exhibit a significantly higher expression of ACE2 compared to non-smokers. Interestingly, Guan et al. (3) reported that both former smokers (49%) and current smokers (21.7%) are at a higher risk of developing severe disease compared to non-smokers (14.5%). Moreover, Cai et al. (11) also observed a significantly higher ACE2 gene expression in the lungs of former smokers compared to non-smokers, as well as a higher expression of ACE2 in current smokers compared to non-smokers. Our results suggested that smokers, especially males, may be more susceptible to infection with COVID-19. With regard to the association between cancer and susceptibility to infection with COVID-19, it has been reported in recent studies that breast, colorectal, and lung cancer may be associated with an increased expression of ACE2 (8, 10). Zheng et al. (16) reported that 8 (1%) of 1,590 COVID-19 cases had a history of cancer, which seems to be higher than the incidence of cancer in the overall Chinese population ([0.29%] per 100,000 people). Liang et al. (17) recently also suggested that patients with cancer might be at a higher risk of infection compared to those without. In this study, we also reported that subjects with cancer exhibit a higher expression of ACE2 compared to those without.

Furthermore, hypertension and cardiocerebrovascular diseases were found to cause a higher expression of ACE2 and, hence, a higher risk of infection. However, multivariate linear regression analysis showed no statistically significantly link between ACE2 and hypertension, ACE2 and cardiocerebrovascular diseases. Moreover, we did not observe any significant difference in the ACE2 gene expression between subjects with diabetes and those without, between drinkers and non-drinkers, and between subjects with different BMI values (<18.5, 18.5–24, 24–27, >27 kg/m^2^). However, Rao et al. (8) showed that diabetes is associated with an increased expression of ACE2. One study (14) showed that, in subjects infected with COVID-19 who developed acute respiratory distress syndrome, compared to those who did not, more patients presented with hypertension (23/84 patients [27.4%] and 16/117 patients [13.7%]) and diabetes (16/84 patients [19.0%] patients and 6/117 patients [5.1%]). Our results are preliminary but are worthy of further studies.

Multiple previous studies have shown ACE2 expression in the lung, kidney, heart, testis, and small intestine of humans (10, 11, 18). As far as we known, blood cells are also the sources of the ACE2 mRNA. For example, Rutkowska-Zapała et al. (19) and Obitsu et al. (20) both reported that ACE2 mRNA was observed in human monocytes and their subsets. Moreover, apoptotic bodies, exosomes, or cast-off cells of endothelial cells contain mRNA that might contribute to the results. Further studies were needed.

In conclusion, we herein identified several demographic and clinical characteristics that may be causally related to the level of ACE2, the level of ACE2 is higher in females, older subjects, smokers, and subjects with cancer than in other subjects. Thus, gender, age, smoking habits, and cancer may provide valuable information for identifying susceptible populations.

## Data Availability Statement

The raw data supporting the conclusions of this article will be made available by the authors, without undue reservation.

## Ethics Statement

The studies involving human participants were reviewed and approved by Eye, Ear, Nose and Throat (Eye and ENT) Hospital of Fudan University, Shanghai, China, and was approved by the Ethics Committee of the same hospital. Written informed consent to participate in this study was provided by the participants' legal guardian/next of kin.

## Author Contributions

SL and WC conceived the study and participated in drafting the final manuscript. SL, JH, AZ, YH, MC, ZL, MS, and WC analyzed the data and completed the final draft of the manuscript. SL and JH prepared all the figures. All authors have read and approved the manuscript.

## Conflict of Interest

The authors declare that the research was conducted in the absence of any commercial or financial relationships that could be construed as a potential conflict of interest.
